# Using Spent Brewer's Yeast to Encapsulate and Enhance the Bioavailability of Sonochemically Nanostructured Curcumin

**DOI:** 10.1155/2024/7593352

**Published:** 2024-11-16

**Authors:** Irina Kalinina, Rinat Fatkullin, Natalya Naumenko, Natalia Popova, Darya Stepanova

**Affiliations:** ^1^Department of Food and Biotechnology, South Ural State University (National Research University), 76 Lenin Avenue, Chelyabinsk 454080, Russia; ^2^Faculty of Biotechnologies (BioTech), ITMO University, Kronverksky Prospekt, 49, lit. A, Saint Petersburg 197101, Russia

**Keywords:** bioavailability, curcumin, encapsulation, nanostructuring, spent brewer's yeast, ultrasound

## Abstract

This study is aimed at investigating the possibility of using spent cells of brewer's yeast *Saccharomyces cerevisiae* to encapsulate the plant antioxidant curcumin and the effect of such an approach on the bioavailability of BAS in an *in vitro* digestion model. Spent brewer's yeast is a significant volume organic waste that is difficult to dispose of, which makes additional options for its use very promising. Encapsulation of curcumin into spent yeast cells was carried out in a nanostructured manner. The encapsulation process was studied using laser dynamic light scattering, inverted and luminescence microscopy, and FTIR analysis. The efficiency and feasibility of curcumin encapsulation process was evaluated by determining the encapsulation efficiency index and modeling the *in vitro* digestion process. From these studies, it was found that spent yeast cells are capable of acting as a “delivery system” for curcumin. Encapsulation efficiencies of 47.7% can be achieved if curcumin is prenanostructured. Analysis of the potential bioavailability of the plant antioxidant in an *in vitro* gastric digestion model showed that the technology of encapsulation into yeast cells allows for curcumin retention of 79.5%.

## 1. Introduction

*Saccharomyces* yeasts are unicellular eukaryotic fungi whose primary mode of reproduction is budding and sometimes fission. To date, about 1,500 species of yeast are known. The yeast *Saccharomyces cerevisiae* is the most utilized microorganism in the food industry. Traditionally used in the production of alcoholic beverages (such as beer, wine, and cider), bakery products, and soft drinks (such as kvass), yeasts are now finding additional applications: they are used in the production of renewable fuels (bioethanol), food ingredients, and pharmaceuticals [1, 2].

The amount of *S. cerevisiae* cells required on an industrial scale can be easily provided, as yeast is a byproduct of large fermentation industries. During fermentation, yeast biomass increases on average 3–6 times. A typical fermentation of lager-type beer produces approximately 2.6 kg of excess dry yeast per cubic meter of beer produced [2]. Unlike the biomass produced in the pharmaceutical industry, yeast cells from fermentation industries are stable and are not subjected to the radical processing associated with the recovery process of the primary product.

Residual brewer's yeast from the beer production process, despite its reuse in the technological process up to six times, represents the second largest volume of waste generated in the brewing industry, accounting for approximately 3% of the volume of beer brewed. Because of its composition, residual brewer's yeast has a high chemical oxygen demand (210,000 mg L^−1^) and biochemical oxygen demand (140,000 mg/L). Additional uses of spent yeast mass are particularly promising also because improper disposal of residual yeast is detrimental to the environment and final disposal imposes additional costs on the industry [2–4].

An alternative use of *S. cerevisiae* cells can be associated with their application as a protective material for biologically active substances in the preparation of functional food ingredients by encapsulation methods [4, 5].

*S. cerevisiae* strains are generally recognized as safe organisms, which increases their attractiveness for the production of food ingredients [4–6].

An important advantage of yeast, especially *S. cerevisiae*, is that it represents a suitable model for basic research. The yeast spp. *S. cerevisiae* are eukaryotic cells that can be easily cultured and manipulated, and they have a fully sequenced genome [1, 4].

The present study involves the utilization of spent yeast mass to encapsulate the plant antioxidant curcumin, which is a promising natural compound used for the prevention and treatment of inflammatory diseases. The curcuminoid curcumin is found in the rhizomes of *Curcuma longa* L. (Zingiberaceae) and has antioxidant and anti-inflammatory properties [7, 8] and is able to modulate the activity of key transcription factors associated with inflammation [8–10]. Curcumin is also used as part of adjuvant therapy to conventional drug therapy for cancer and a number of pathologic conditions. Several studies have described the ability of curcumin to exhibit the properties of a neuroprotective molecule. The antioxidant has been tested in several brain diseases, including Huntington's disease, an inherited neurodegenerative condition caused by the expansion of the polyglutamine tract in the huntingtin protein [8, 9, 11].

Although curcumin exhibits a large number of pharmacological effects under in vivo conditions, it has very low bioavailability under real-world consumption conditions, which poses a major challenge to its wider use [9, 10]. The low oral bioavailability of curcumin is caused by poor absorption in the small intestine due to its low water solubility, rapid metabolism in the liver, and rapid excretion [7, 9, 10].

One of the directions for improving the delivery of curcumin to target cells may be the use of technologies for its encapsulation in micro- and nanostructured form. Modification of curcumin aimed at reducing the particle size of the antioxidant allows for improving its solubility and bioavailability. A number of studies have shown the possibility of improving the properties of curcumin by high-pressure treatment [11], microfluidization, solid dispersion method, and Fessy method [12]. One of the alternative and effective approaches for nanostructuring of curcumin is sonochemical treatment under cavitation exposure conditions. Ultrasound has been widely used in recent years in various areas of research, such as biotechnology, medical, and food technology. The cavitation effect in liquid media is due to the formation of cavitation bubbles, the destruction of which generates excess pressure waves, which make it possible to reduce the size of curcumin particles.

Curcumin encapsulation technologies are widely presented in the literature using various encapsulation coatings: gelatin, cyclodextrins, cationic micelles, liposomes, and modified starch [13, 14].

A number of studies on the encapsulation of curcumin into live yeast cells, into plasmolyzed yeast, and into yeast cell walls have also been presented. The achieved encapsulation efficiencies are highly subject to fluctuations ranging from 20% to 60% and are primarily determined by the encapsulation method [10, 15, 16].

Meanwhile, the use of curcumin pretreatment, in particular ultrasonic nanostructuring, to improve the encapsulation efficiency index (EI) has not been described in studies, which determines the novelty of the present work.

**Figure 1:**
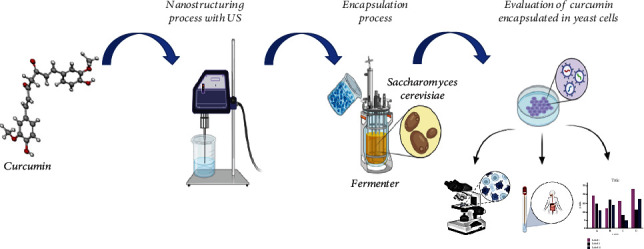
Generalized scheme of research.

The present work is aimed at studying the possibility of using the spent cells of brewer's yeast *S. cerevisiae* as a carrier for encapsulation of nanostructured curcumin and to evaluate the potential bioavailability of the encapsulated antioxidant (Figure 1).

## 2. Materials and Methods

### 2.1. Encapsulation Conditions

#### 2.1.1. Nanostructuring of Curcumin

Curcumin was encapsulated in a nanostructured form. The nanostructuring process was carried out using an immersion-type ultrasonic apparatus according to our previously developed method described in [17, 18]. Briefly, a 0.1% (*w*/*v*) mixture of curcumin with distilled water in a total volume of 100 mL was ultrasonicated using the previously established mode: 630 W/100 mL for 10 min. Nanostructured curcumin powder was obtained after freeze-drying.

#### 2.1.2. Curcumin Encapsulation Technology

The technology of curcumin encapsulation in yeast cells included mechanical mixing of the components in an aqueous medium in a weight ratio of 1:1 (yeast:curcumin) at 28°C, 36°C, and 44°C for 24 h. The process was monitored every 2 h. The choice of these temperature conditions is due to the need to ensure the phase transition of the phospholipids of the yeast cell wall membranes from the gel state to the liquid and liquid-crystalline state, which will ensure better penetration of curcumin into the yeast cells.

### 2.2. Nomenclature of Parameters and Methods of Analysis

#### 2.2.1. Evaluation of Properties of Nanostructured Curcumin

The morphological structure of curcumin samples in native and nanostructured form was obtained using an inverted microscope (Altami INVERT 3), magnification ×600.

The disperse composition was determined by laser dynamic light scattering using Microtrac NANOTRAC FLEX.

Commercially available *S. cerevisiae* yeast (strain W68), spent after brewing (more than 90% dead cells), were used as research objects. Curcumin was purchased from Nanjing Duly Biotech Co., Ltd., China (at least 95% purity).

#### 2.2.2. Evaluation of Encapsulation Efficiency

To evaluate the efficiency of the encapsulation process of nanostructured curcumin into spent yeast cells, the values of two indices were established [19]:

The amount of biologically active ingredient that penetrated the yeast cells and adhered to the surface of the yeast cells

The amount of biologically active substance remaining in the solution

To determine the value of indicator (ii), the suspension obtained after the encapsulation process was centrifuged at 5,000 rpm for 2 min and the curcumin content of the supernatant was determined

To establish the value of indicator (i), 10 mL of ethanol and 10 mL of 5N HCl were added to the 0.1-g thick yeast mass obtained after centrifugation and mixed vigorously on a shaker (Vortex) until the capsules were broken. Then centrifuged at 5,000 rpm and the curcumin content of the supernatant was determined

The curcumin content was determined spectrophotometrically using Folin–Ciocâlteu reagent by measuring the optical density at 700 nm. A calibration plot for curcumin was preliminarily constructed using the state standard reference sample.

EI in percentage was calculated using the following formula:

(1) $$\begin{array}{c} \text{EI }\left(\%\right)=\left[\frac{\left(\text{X}1-\text{X}0\right)}{\text{X}2}\right]\times 100, \end{array}$$

where X1 is the amount of biologically active substance that penetrated inside yeast cells and adhered to the surface of yeast cells (i), mg; X0 is the amount of biologically active substance not encapsulated, remaining in the solution (ii), mg; and X2 is the total amount of BAS added during encapsulation, mg.

#### 2.2.3. Study of Encapsulation Mechanism

Microphotographs of the encapsulated curcumin were obtained using Micromed 3 Alpha fluorescence microscope, magnification ×600.

Fourier transform infrared spectroscopy (FTIR) studies were also performed (Shimadzu Iraffinity-1S spectrophotometer was used) using c KBr disk method. Spectra in the range of 400–3900 cm^−1^ were taken for lyophilized samples of curcumin encapsulated in yeast cells, plasmolyzed empty yeast, and nanostructured curcumin. Triplicate determinations were performed for each sample.

#### 2.2.4. Evaluation of Bioavailability of Curcumin Encapsulated in Yeast Cells

The potential bioavailability was estimated based on the determination of bioavailability index (BAI) according to the methodology in [16] which briefly includes *in vitro* digestion modeling in the gastric phase using appropriate pH system, gastric juice, bile salts, and pepsin enzyme. The amount of curcumin was determined in the obtained digested fraction after 30, 60, 90, and 120 min.

BAI (%) is calculated according to the following formula:

(2) $$\begin{array}{c} \text{BAI}=\left(\text{Cfinal}\right)/\text{Cinitial}\times 100, \end{array}$$

where Cfinal is the concentration of BAS after *in vitro* cleavage process and Cinitial is the concentration of BAS in the tested solution before the cleavage process.

Extraction and quantification of curcumin was carried out as follows. Curcumin was extracted with 1 mL of methanol from 0.01 g of yeast microcarrier (in terms of crude weight). The samples were then shaken, treated in an ultrasonic bath for 10 min, and centrifuged at 16 × 100*g* for 10 min. The amount of curcumin was determined spectrophotometrically with aluminum chloride. The optical density of the solution was determined on a spectrophotometer at a wavelength of (425 ± 2) nm.

## 3. Experimental Results and Their Discussion

### 3.1. Investigation of the Properties of Nanostructured Curcumin

Analyzing the data on the chemical composition and key properties of curcumin as a biologically active substance, we can note its extreme hydrophobicity (solubility in water does not exceed 0.001%), close to the critical value of lipophilicity, log *p* = 3.6, a sufficiently large number of hydrogen acceptors (equal to 6) and a significant molecular mass (368.38) [20].

With such characteristics, the full manifestation of the biologically active properties of curcumin in real conditions of its consumption seems very doubtful. Complicating the task is the tendency of curcumin molecules to polymerization, which together dictates the need to search for technologies to modify polyphenol to change its properties.

**Figure 2:**
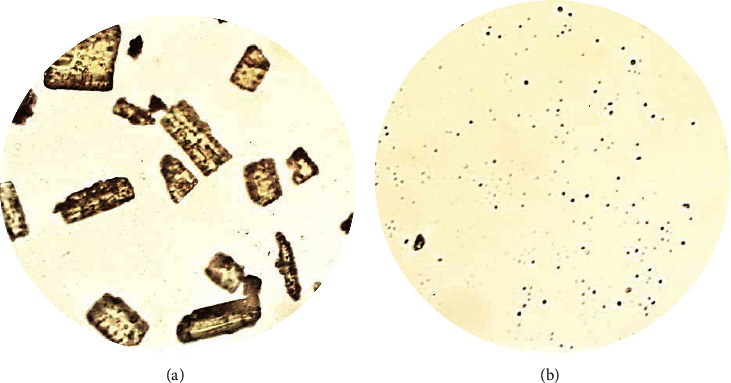
Microscopy of curcumin solution (hanging drop preparation, magnification ×600, inverted microscope): (a) curcumin ex. and (b) curcumin USV 630 W 10 min.

**Figure 3:**
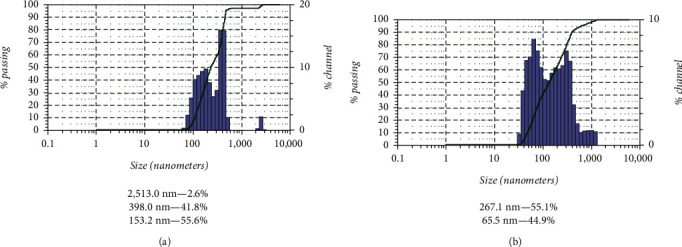
Results of dispersion analysis of curcumin solutions: (a) control and (b) nanostructured (ultrasonic 630 W/L, 10 min).

To correct the properties of curcumin, we used ultrasonic nanostructuring technology, which allowed us to change the dispersed composition and morphology of curcumin particles, which is confirmed by the microphotographs presented in Figure 2 and the results of the dispersed composition evaluation (Figure 3).

The photographs presented in Figure 2 demonstrate that ultrasonic treatment can reduce the size of curcumin particles many times. Large, irregularly shaped crystals are converted to a fine crystalline state close to amorphous state during nanostructuring.

Ultrasonic treatment of aqueous suspension of biologically active substance, in our case, curcumin, was aimed, first of all, at depolymerization of its particles. It is known that ultrasonic impact in cavitation mode allows to form the effect of collapse of cavitation bubbles, as a result of which zones of overpressure appear in the liquid system [18, 21–25]. In fact, the action of the “blast wave” and the formed vortex flows as a result of cavitation leads to the formation of smaller and more homogeneous in size particles.

Analysis of the disperse composition showed that the particle size of the initial curcumin is scattered in a very wide range, 153.2–2513 nm (Figure 3(a)). In addition, given the pronounced hydrophobicity of curcumin, the possible presence of particles significantly larger than 2500 nm cannot be excluded. Such particles could be beyond the sensitivity of the instrument.

Ultrasonic nanostructuring of curcumin allowed us to achieve a significant reduction in the range of values of its particle size. Analysis of the disperse composition showed the predominance of two size ranges −267.1 and 65.5 nm (Figure 3(b)).

The reduced size of curcumin suggests an easier option for its penetration into the yeast capsule. Since many studies indicate that the process of encapsulation of bioactive substances into yeast carriers proceeds predominantly by diffusion processes, the size of curcumin particles becomes essential for the efficiency of the encapsulation process [4, 6, 10].

Increasing the contact area of small curcumin particles with the solvent allows improving such an important property for encapsulation technology as water solubility [17, 18, 20, 21]. Yeast is known to be able to sorb hydrophobic compounds as well. However, given that the encapsulation process was carried out in aqueous medium, improved solubility of curcumin in water is necessary.

The process of sonochemical nanostructuring of curcumin allowed achieving a particle size of the encapsulated substance that is several times smaller than the size of the delivery system—yeast cells. This gives grounds to assume a higher level of loading of curcumin into yeast cells, without changing the size of the latter.

### 3.2. Evaluation of the Encapsulation Efficiency of Curcumin Into Plasmolyzed Yeast Cells

**Figure 4:**
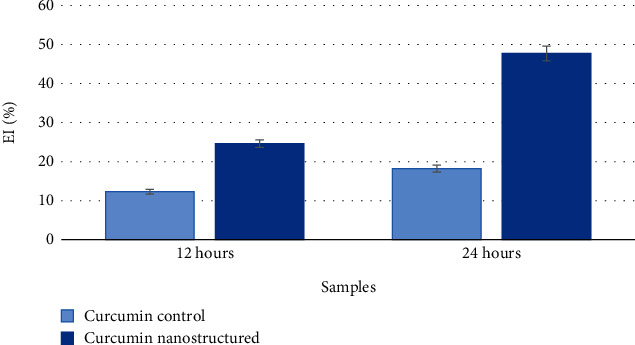
Results of determining the encapsulation efficiency of curcumin in its original and nanostructured form into yeast cells, in percentage.

To test the hypothesis of the positive effect of the ultrasonic nanostructuring process of curcumin on the efficiency of its encapsulation into yeast cells, we evaluated the EI (Figure 4).

The results showed a twofold increase in efficiency values with the nanostructured form after 12 h and a 2.6-fold increase after 24 h compared to the original form.

The explanation for this fact may be a change in the disperse composition of curcumin under the action of ultrasound and, as a consequence, its higher solubility in water and permeability through the cell membranes of yeast cells. The yeast cell contains a huge number of components with functional groups. The main dry mass of yeast cell wall is 80%–90% composed of polysaccharides, proteins, and lipids such as glucans, chitin, mannans, and phosphomannans [26–28] with functional groups such as hydroxyl, carbonyl, chitin, acetamide, carboxyl, sulfhydryl, thioether, sulfonate, amine, amide, imidazole, phosphonate, and phosphodiester. They are the ones that act as a source of ligand for the formation of complexes with encapsulated substances and retain them in the biomass [27, 29–32].

However, there are other ways of encapsulation of organic substances into the yeast cell, including such processes as intracellular accumulation and extracellular deposition, which are metabolically independent [8, 27, 30]. The mechanism of small molecule encapsulation by the cell surface of inactivated yeast cells includes physical adsorption, chelation, electrostatic interactions, and complexation [19, 30–32]. These are metabolically independent biosorption processes based on physicochemical interactions between the encapsulated molecule and functional groups present on the yeast cell surface. The pathway used for surface sorption due to nonspecific attraction forces such as van der Waals forces is physical adsorption, which is rapid and reversible.

According to some data, biosorption of organic substances by yeast can be represented by the following stages: migration of organic compounds from liquid solution into the boundary layer of yeast particles; surface diffusion inside yeast cells; diffusion into biomass pores; and biosorption of active centers inside cells [25, 30, 32]. Each of these steps is characterized by its own values of specific rate, which in general affects the EI.

**Figure 5:**
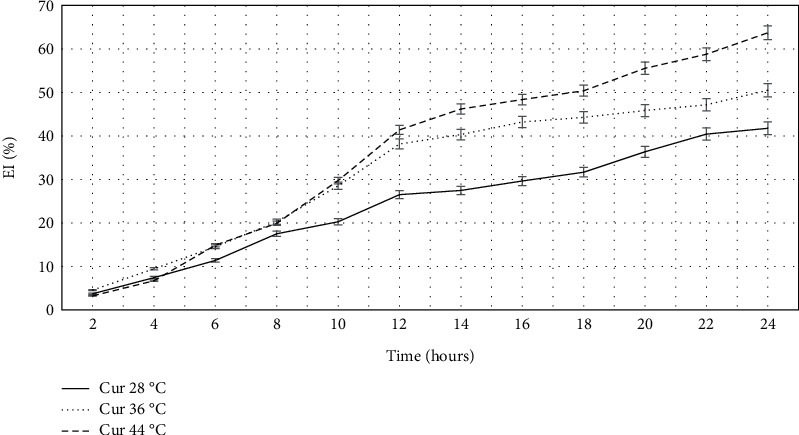
Dynamics of the process of encapsulation of curcumin samples into yeast cells.

Evaluation of the kinetics of the encapsulation process of curcumin in nanostructured form into spent *S. cerevisiae* yeast cells showed that both the duration of the encapsulation process and the temperature contributed significantly to the overall process EI (Figure 5).

Thus, at the 24-h control point, the EI was on average 1.3–1.5 times higher than at the 12-h mark, regardless of the encapsulation temperature used. The positive effect of the duration of the encapsulation process on its efficiency was expected and is in agreement with the literature [15, 16]. Among all possible factors affecting this course of the process, two should be highlighted: the increase of dissolved curcumin over time and its gradual penetration into the cells of plasmolyzed yeast. The use of a temperature of 44°C resulted in an increase in the efficiency of encapsulation after 24 h by about 1.5 times relative to 28°C. This may be due to the need to ensure a sufficiently viscous-liquid state of the yeast cell wall membrane as the main barrier for curcumin penetration into the cell. The obtained results once again confirm the assumption of a significant contribution of diffusion processes to curcumin encapsulation.

It was found that, in general, the temperature of the encapsulation process did not significantly affect the general character of the process kinetics. In the first 6 h, the encapsulation process proceeded with rather low intensity. The most active period was noted for time intervals of 6–12 and 18–24 h.

Compared to other encapsulation approaches, nanostructured curcumin yeast capsules showed remarkable and comparable %EE values. A %EE value of 32.04 was reported for curcumin-phospholipid complex which was prepared with curcumin and hydrogenated soy phosphatidylcholine in a 1:1 molar ratio. Furthermore, encapsulation of curcumin in gelatin using simple coacervation method resulted in an increase in %EE to 75.53 [13]. In contrast, the %EE of curcumin nanoparticles in alginate and chitosan polymers was found to be around 13 [14].

### 3.3. Analysis of Encapsulation Mechanisms

**Figure 6:**
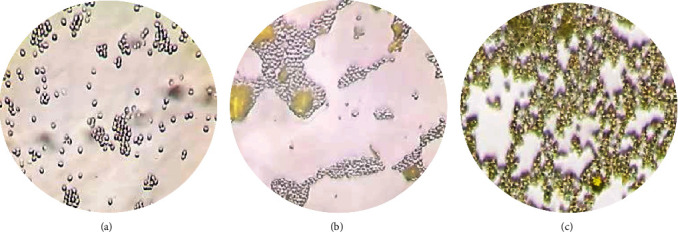
Microscopy of curcumin solution (magnification ×600, fluorescent microscope): (a) yeast ex. blank; (b) physical mixture of yeast and curcumin; and (c) nanostructured curcumin encapsulated in yeast cells.

To confirm the encapsulation of curcumin in yeast cells, fluorescence microscopy was used in the present study. Curcumin is known to have fluorescence properties, so its presence can be assessed using fluorescence microscopy (Figure 6).

Comparative analysis of microphotographs allows us to assert that curcumin is mainly contained inside yeast cells. At the same time, the analysis of several fields of view showed the presence of single rather large curcumin particles on the surface of yeast cells, indicating incomplete penetration of the biologically active substance into yeast cells.

**Figure 7:**
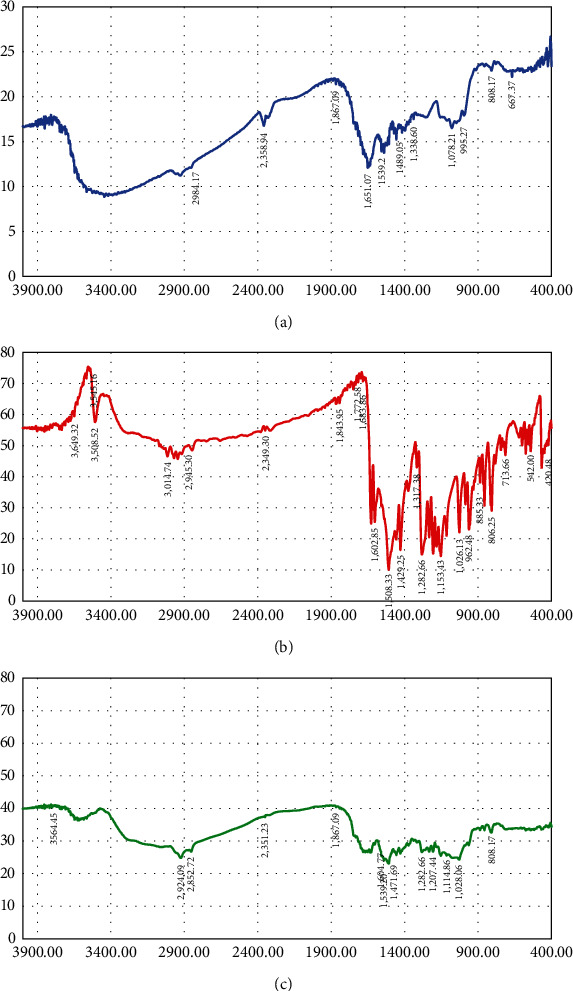
FTIR spectra of the studied samples: (a) plasmolyzed yeast, (b) nanostructured curcumin, and (c) nanostructured curcumin encapsulated in plasmolyzed yeast.

FTIR spectral analysis was used to analyze the possible conformational interactions of curcumin with yeast cell structures (Figure 7).

According to literature data, when curcumin interacts with yeast cell components with the occurrence of noncovalent interactions between them (van der Waals interactions, hydrophobic interactions, hydrogen bonds), the energy of the part of curcumin that participates in the interactions decreases and, consequently, its absorption frequency decreases or the peak intensity in the corresponding region changes [10]. In addition, the possible shift of characteristic absorption bands of yeast cells may also provide evidence for the interaction between curcumin and yeast cell components.

The IR spectrum of nanostructured curcumin encapsulated in yeast was similar to that of empty yeast cells, whereas the IR absorption bands of curcumin, the sharp peaks decreased significantly, almost disappearing. This observation suggests that the main curcumin bands were attenuated when they interacted with yeast cell components, and that curcumin molecules are rather located inside yeast cells, so their IR signature was “hidden.”

The IR spectrum of the encapsulated form of curcumin showed the disappearance of the peak in the 3,500-cm^−1^ region, evident in the spectrum of pure curcumin, indicating that the phenolic −OH groups interacted with yeast cell components, most likely through hydrogen bonding, while the smoothing of the 1,600-to-950-cm^−1^ band indicated that its aromatic rings also interacted with yeast cell components.

There was an increase in the intensity of the peaks of encapsulated yeast compared to pure yeast in the region 2,960–2,930 cm^−1^, demonstrating the interaction of curcumin with yeast phospholipids. Differences in the absorption bands at 1,660–155 cm^−1^ between empty yeast cells and encapsulated cells indicate the interaction of curcumin with cell wall protein, whereas changes in the 1,100-to-1,000-cm^−1^ region suggest an interaction between curcumin and glucans. Changes in the 1,200-to-1,250-cm^−1^ region may indicate penetration of curcumin into the yeast cell.

In general, it can be assumed that when encapsulated in yeast cells, curcumin penetrates into the cells and participates with the occurrence of hydrophobic interactions with densely packed hydrocarbon chains of membrane phospholipids. At the same time, the presence of a hydroxyl group in the polyphenol molecule increases diffusion across the plasma membrane. The hydroxyl or any amphiphilic group is attracted and stabilized by hydrogen bonds with polar head groups of membrane phospholipids, and, as a consequence, the contact between the hydrophobic part of the molecule and acyl chains increases.

### 3.4. Evaluation of Bioavailability of Encapsulated Curcumin

The main task of encapsulation of biologically active substances is their protection from external destructive effects, especially during gastric digestion and delivery of the maximum amount of BAS to the small intestine, from where the substance can enter the systemic bloodstream [19, 33–36]. Encapsulation gives the active compound some degree of stabilization, since the material of the protective capsule acts as a physical barrier to mechanical destruction; to oxygen, preventing oxidative processes; and to enzymes and acids, which in the process of digestion prevents premature incorporation of the active substance into metabolic processes [34, 37–39]. At this stage of research, to evaluate the effectiveness of the selected approaches, an attempt was made to study the role of technologies for encapsulation of nanostructured curcumin into yeast cells for the amount of polyphenol in the process of simulated digestion in the stomach.

**Figure 8:**
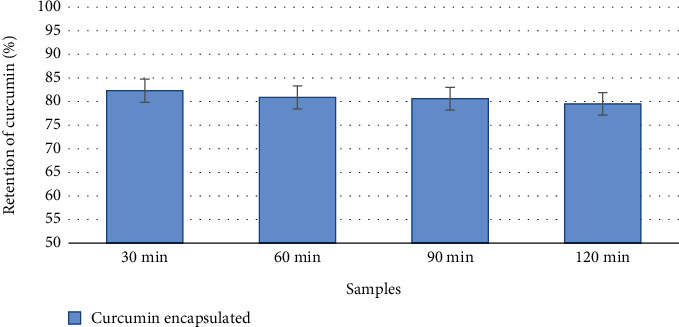
Potential bioavailability of curcumin encapsulated in yeast cells.

Considering encapsulation processes in terms of their influence on the biological effects of the formed complexes, most studies prove that encapsulation technology promotes the preservation of polyphenols during digestion, ensuring their more efficient delivery to the human body [35, 37, 40–43]. According to the literature, quantitative degradation as a result of *in vitro* digestion depends primarily on the class of the phenolic compound, its resistance to pH, enzymes, and propensity for structural transformations that lead to metabolites with different chemical properties and generally lower bioactivity [41, 44–48]. Studies have shown that encapsulation of curcumin in spent yeast cells after *in vitro* gastric digestion procedure allows preservation of more than 80% of BAS (Figure 8).

About 18% of curcumin is released during the first 30 min of digestion; thereafter, we did not observe statistically significant changes in the amount of encapsulated curcumin. Probably, the losses of encapsulated curcumin detected during digestion are related to the destruction of BAS remaining (stuck) on the surface of the yeast cell wall.

A number of studies on the yeast cell digestion process show a fairly good resistance of cell wall mannoproteins to the action of pepsin and gastric juice, which allows for a limited release of curcumin bound to cell wall biostructures (mannoproteins and beta-glucan) during the gastric digestion phase [27–29]. The yeast cell is subjected to stronger degradation under the influence of intestinal bile acids. This leads to a more active release of the BAS encapsulated in the yeast cell precisely at the stage of intestinal digestion. More effective delivery of curcumin encapsulated in yeast cells is probably due to the fact that part of the curcumin binds to the hydrophobic regions of the biopolymers of the intracellular structures of the yeast cell, which is confirmed by the FTIR analysis data. In this case of interaction, the encapsulated substance does not destroy the yeast cell wall, which in turn allows maintaining a balance between the permeability of the yeast cell wall and the ability to preserve the penetrated substance of the nucleus. The encapsulation properties of yeast cells are the synergy of proteins, nucleic acids, lipids, fatty acids, and polysaccharides [27–30].

These results show that the nanostructured curcumin encapsulated in spent yeast cells had a good effect of prolonged release of antioxidants under simulated digestion conditions, which is comparable or slightly superior to the results of similar studies [47].

## 4. Conclusion

Thus, the conducted studies confirmed the possibility of encapsulation of curcumin into spent yeast cells of *S. cerevisiae* by simple diffusion. Using the technology of prenanostructuring of curcumin, it is possible to increase the efficiency of its encapsulation into yeast cells by 2.6 times compared to the original form. The obtained micrographs confirmed the penetration of the biologically active substance inside yeast cells. The results of FTIR analysis suggested that among the most probable interactions of curcumin with structural elements of the yeast cell, interaction with proteins, polysaccharides, and phospholipids of the yeast wall can be identified. The study of yeast cell effects on the potential bioavailability of curcumin demonstrated high protective properties of yeast cells against the damaging effects on curcumin of gastric digestion processes. In the first 30 min, about 18% of the BAS was released, which may be due to the destruction of particles remaining on the surface of the yeast cell wall. Further studies may be required to better understand the processes of encapsulation of bioactive substances into yeast cells and to describe the mechanism of delivery of encapsulated bioactive substances. Further search for ways to increase the efficiency of encapsulation while shortening the duration of the process is necessary. It is important to understand the role of individual factors in the digestion process on efficiency.

Thus, future studies may focus on optimizing curcumin encapsulation parameters to maximize the stability of delivery systems and antioxidant release profiles. For example, the effects of pH and bile acid concentration may influence the stability and release mechanisms of curcumin encapsulated in yeast cells. In addition, the safety of these materials should be carefully evaluated, especially in terms of their long-term biodegradability and potential toxicity. This will not only ensure compliance with regulatory standards but also increase consumer acceptance, especially in the food and medical industries.

## Data Availability

Data will be made available upon request to the corresponding authors.
